# Nonunion of a Third Metatarsal Base Stress Fracture Injured During Cross-Country Skiing Treated by Osteosynthesis: A Case Report

**DOI:** 10.7759/cureus.79407

**Published:** 2025-02-21

**Authors:** Yusuke Yoshimoto, Hidenori Matsubara, Toshifumi Hikichi, Kanu Shimokawa, Satoru Demura

**Affiliations:** 1 Orthopaedic Surgery, Kanazawa University Hospital, Kanazawa, JPN; 2 Orthopaedic Surgery, Kaga Medical Center, Kaga, JPN

**Keywords:** cross-country skiing, nonunion, osteosynthesis, stress fracture, the third metatarsal

## Abstract

Stress fractures at the base of the metatarsal are classified as high-risk stress fractures. The second and third metatarsal are likely to occur in classical ballet and jumping sports. Among them, there are few reports of nonunion cases after fractures at the base of the third metatarsal. We present a rare case of nonunion of a stress fracture at the base of the third metatarsal injured during practicing cross-country skiing. A 15-year-old male high school top-level cross-country skier couldn’t stand the pain on the dorsum of his left forefoot during training for six months. Radiographic examination and computed tomography showed a transverse fracture line at the base of the third metatarsal and an osteosclerotic lesion at the fracture site. He was diagnosed with nonunion of a stress fracture at the base of the third metatarsal. We performed osteosynthesis with a locking plate and grafted autogenous bone into the nonunion site. He was able to return to skiing at 4 months postoperatively. At 2.5 years after the initial surgery, he was able to participate in an international cross-country skiing competition. We experienced nonunion following a stress fracture at the base of the third metatarsal, possibly injured while practicing cross-country skiing. Osteosynthesis with the plate fixation technique using autogenous bone grafting was a useful treatment for the nonunion of a stress fracture at the base of the third metatarsal.

## Introduction

Metatarsal stress fractures tend to occur in the second and third metatarsal shaft, and metatarsal shaft fractures are classified as low-risk stress fractures with a good prognosis with conservative treatment [1]. Conversely, stress fractures at the base of the metatarsal are classified as high-risk stress fractures, and cases of nonunion and delayed union have been reported [1]. Among them, there are a few cases of stress fractures at the base of the second and third metatarsal, which usually occur at the metatarsal shaft [2]. Kameyama reported that the incidence of third metatarsal stress fractures is 27.9% of all metatarsal stress fractures, but the base of the third metatarsal stress fractures is rare: 57 cases at the shaft and 2 cases at the base [2]. As a background of injury, stress fractures at the second and third metatarsal bases are likely to occur in classical ballet and jumping sports [3,4]. However, there are only a few reports of stress fractures of the base of the metatarsal caused by cross-country skiing, and there are also a few reports of surgical treatment of nonunion cases. We present nonunion following a stress fracture at the base of the third metatarsal, possibly injured during practicing cross-country skiing, and consider the mechanism of injury.

## Case presentation

**Figure 1:**
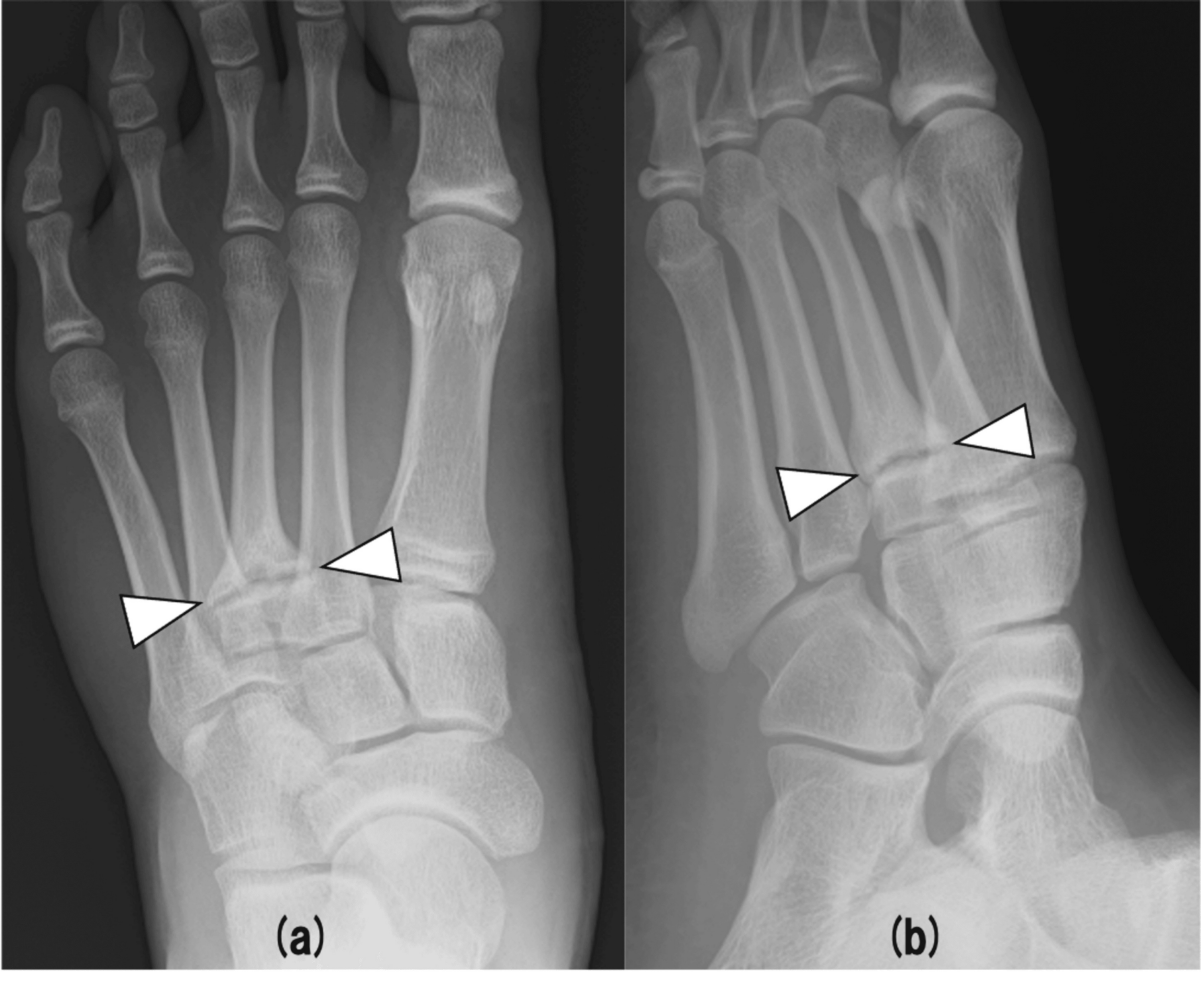
Preoperative radiographs A transverse line of fracture was identified on anterior-posterior (a) and oblique (b) views.

**Figure 2:**
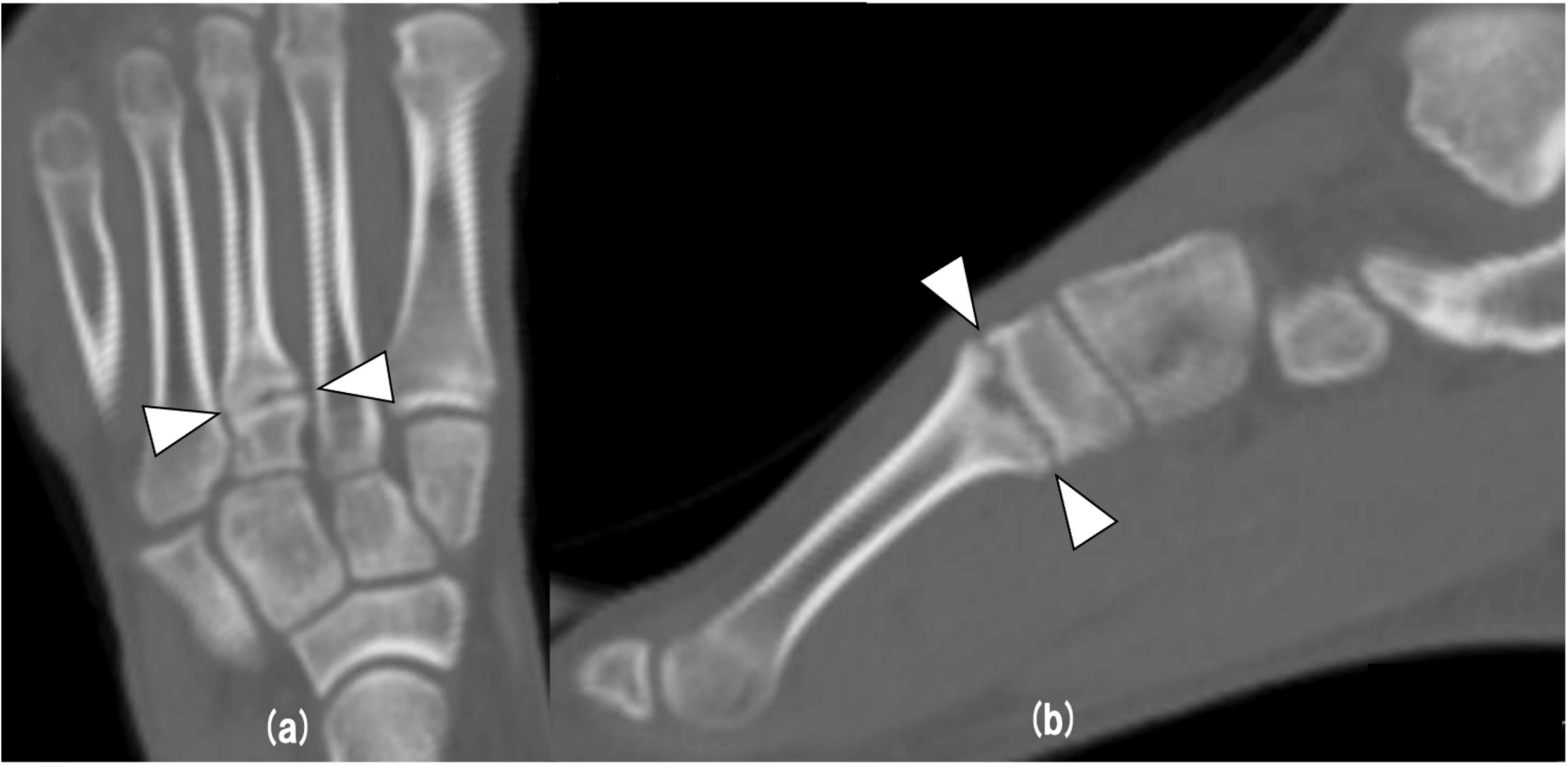
Preoperative CT Preoperative CT showed the line of fracture at the base of the third metatarsal and sclerotic change and irregularity in the region of the fracture site on coronal (a) and sagittal (b) views.

**Figure 3:**
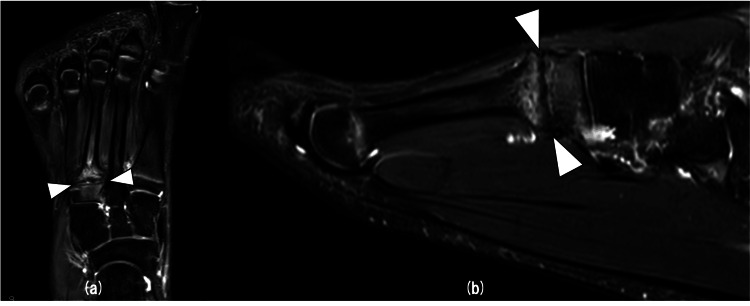
Preoperative MRI T2 fat-suppressed images Preoperative MRI T2 fat-suppressed images demonstrated bone marrow edema in the region of the fracture site in coronal (a) and sagittal (b) views.

A 15-year-old male high school top-level cross-country skier couldn’t stand the pain on the dorsum of his left forefoot while practicing cross-country skiing. After six months, the pain had not improved, so he visited a neighborhood clinic. The clinic doctor diagnosed the patient with nonunion of a stress fracture at the base of the third metatarsal. He was referred to our hospital for further management. On physical examination, there was no swelling of the dorsum of his left foot but tenderness at the base of the left third metatarsal. The range of motion of the right forefoot inversion was 45°, and eversion was 10°, and that of the left forefoot inversion was 50° and eversion was 5°. AOFAS (American Orthopaedic Foot and Ankle Society) midfoot scale was 87 points. Radiographic examination showed a transverse line of fracture at the base of the third metatarsal and osteosclerotic lesion in the region of the fracture (Figure 1). Computed tomography (CT) showed the line of fracture in the same area and sclerotic change and irregularity in the region of the fracture site (Figure 2). Magnetic resonance imaging (MRI) T2 fat-suppressed images showed bone marrow edema around the fracture site in coronal and sagittal views (Figure 3). Based on these findings, we decided to perform osteosynthesis with autogenous bone grafting for nonunion of a stress fracture at the base of the third metatarsal.

Surgical technique

**Figure 4:**
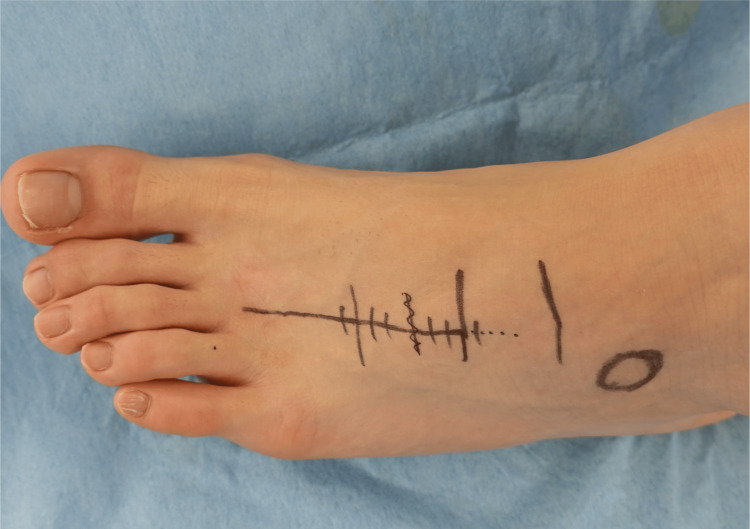
Intraoperative photo The skin incision was placed above the left third metatarsal bone.

**Figure 5:**
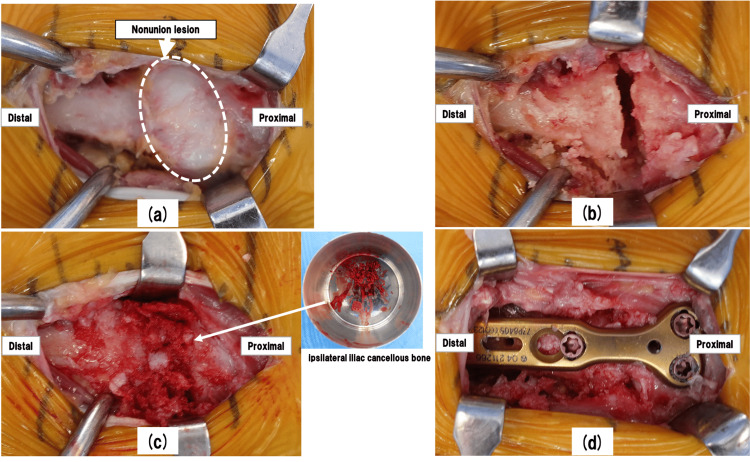
Intraoperative photos

**Figure 6:**
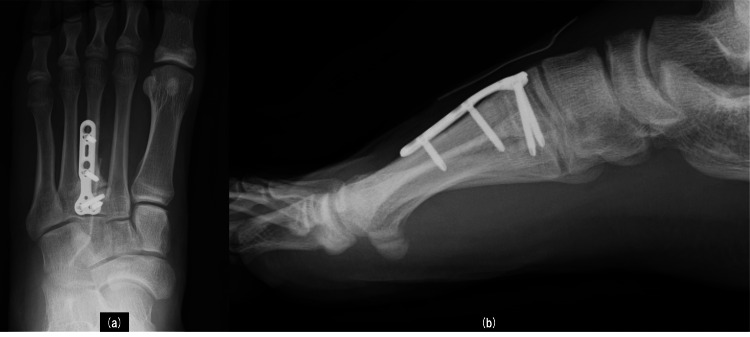
Postoperative radiographs Postoperative radiographs show that plate fixation was performed. Anterior-posterior (a) and lateral (b) views.

The skin incision was placed above the left third metatarsal bone (Figure 4). The nonunion lesion was covered with scar tissue (Figure 5a). The nonunion lesion was refreshed by curettage and multiple drilling with Kirschner-wire (Figure 5b). After that, we collected an autogenous cancellous bone from the ipsilateral iliac crest and then grafted it into the nonunion site (Figure 5c). Subsequently, We performed the plate fixation. The locking plate was placed between the proximal fragment and the distal fragment of the metatarsal bone (Figures 5d-6).

Postoperatively, casting was not used to immobilize the foot. Low-intensity pulsed ultrasound (LIPUS) was used at 3 weeks postoperatively. Partial weight-bearing with forefoot non-weight bearing was allowed at 6 weeks postoperatively with an insole. Full weight bearing was started at 8 weeks postoperatively. The exercise intensity was gradually increased according to the pain, and he was able to return to skiing at 4 months postoperatively. We confirmed complete bone union at 5 months after the operation. He had no symptoms, and the AOFAS midfoot scale was 100 points at 1 year postoperatively. He was able to participate in an international cross-country skiing competition at 2.5 years postoperatively.

## Discussion

The most common overuse injuries in cross-country skiing are medial-tibial stress syndrome, Achilles tendon problems, and lower back pain [5]. The most common traumatic injuries were ankle ligament sprains and fractures, muscle ruptures, and knee ligament sprains [5]. Morris and Hoffman reported that more than half of the traumatic injuries in cross-country skiing were to the lower extremities [6]. Moreover, Rotllan and Viscor reported the frequency of injury in cross-country skiing was 2.7 per 1000 h of practice [7]. The injury prevalence was 21.4% in cross-country skiing, and the most common new injury site was the foot.

In cross-country skiing, the range of motion of the toes and ankle must be unrestricted. The bindings fix the forward tip of the boot while leaving the heel free to elevate from the ski [5]. The “kick” phase in cross-country skiing involves plantar flexion at the ankle. An excessive loading of the tarsometatarsal joint in the plantarflexion position leads to stress fracture [3,8]. In this case, he is thought to have had repeated ankle plantarflexion positions. Due to this motion, the stress is concentrated in the Lisfranc joint, and it is thought to have caused a stress fracture at the base of the third metatarsal. In other words, stress fractures of the base of the third metatarsal may be one of the diseases to watch out for in cross-country skiers.

The risk factors at the base of the metatarsal stress fracture are a contracture of Achilles, shorter first metatarsal length, trauma history of stress fracture, sports (especially classical ballet), and overuse [9].

There are a few cases of stress fractures at the base of the second and third metatarsal reported in the literature [2]. Classical ballet and aesthetic sports are most likely to cause stress fractures at the base of the second and third metatarsal [4]. There is a report that stress fractures of metatarsal are common in military recruits with leg-length discrepancy [1]. Saxena et al. reported a stress fracture at the base of the fourth metatarsal that may have been injured while training for cross-country skiing [10]. As far as we know, there are no reports about the third metatarsal base stress fractures in cross-country skiers.

The diagnosis of stress fracture at the base of the metatarsal is usually nonspecific and can be easily delayed diagnosis or overlooked [3]. Stress fractures are common injuries, but it is often difficult to detect the findings on radiographs, especially in the early course [11]. The gold standard for accurate diagnosis is MRI and scintigraphy. Recently, there have been some articles that ultrasonography is useful for the diagnosis of metatarsal stress fractures because of its low cost, non-invasive, rapid, and easy technique [12]. In this case, he had been suffering from forefoot pain for more than 6 months. Therefore, the diagnosis was easy to make because the fracture line and osteosclerotic lesion were observed on the initial radiograph at a point coinciding with the tender area.

Stress fractures at the base of the metatarsal have been shown to have good results with early detection and strict conservative treatment [8]. In fresh cases, conservative treatment is generally recommended as the initial treatment. The exercise should first be restricted, and an insole should be used for management. If the pain is severe, casting and crutches are considered to be used. Previous reports have shown that the success rate of conservative treatment was 75 to 100% [3,13,14,15]. However, there are reports of treatment failure, such as delayed union and nonunion, because of delays in diagnosing the fracture [10]. Surgical treatment is generally indicated for these treatment failures. Morio et al. reported osteosynthesis with the bridging plate fixation technique [16]. Bridging plate fixation may be useful in cases where the proximal fragment is small. They removed the plate at 12 weeks postoperatively [16]. In the bridging plate fixation, additional surgery is required to remove the metal. Since the plate can help to prevent re-fracture, it is desirable to place a plate within the metatarsal range. Moreover, return to sports is after metal removal. There are several reports of good results with bone grafting in the nonunion and fixation of the proximal and distal bone fragments within the metatarsal range using a locking plate [9,15]. In the present case, the proximal fragment was of an adequate size and could be fixed without crossing the joint. We could confirm complete bone union 5 months after the operation, and he could return to sports without metal removal. His symptoms resolved, and he was able to join international competition without re-fracture.

## Conclusions

We presented the nonunion of a third metatarsal base stress fracture in a top-level cross-country skier. Stress fractures of the base of the third metatarsal may be one of the diseases to watch out for in cross-country skiers. Surgical treatment using a plate fixation with bone grafting has been markedly successful, and he was able to participate in an international cross-country skiing competition.
